# Lipofibroadenoma of the thymus: a case report

**DOI:** 10.1186/1746-1596-8-117

**Published:** 2013-07-15

**Authors:** Guimei Qu, Guohua Yu, Qian Zhang, Junjie Ma, Xiaolei Wang

**Affiliations:** 1 Department of Pathology, Affiliated Yantai Yuhuangding Hospital, Medical College of Qingdao University, No.20, Yuhuangding East Road, Yantai 264000, PR China; 2 Department of Pathology, Binzhou Medical University, No.346, Guanhai Road, Yantai 264003, China; 3 Department of Hametolgoy, Affiliated Yantai Yuhuangding Hospital, Medical College of Qingdao University, No.20, Yuhuangding East Road, Yantai 264000, China

**Keywords:** Lipofibroadenoma, Thymic tumor

## Abstract

We observed an unusual case of Lipofibroadenoma (LFA) in the anterior mediastinum with a 21-year-old man, who was detected with a mass on a chest X-ray scan for one month. Thymothymectomy was then performed and the mass was excised completely, in which the tumor was histologically composed of epithelial cells, lymphocytes, mature adipose and fibrous tissue. Within the tumor, the fat cells was distributed singly or multifocally under the ground of fibro tissue with hyaline degeneration, and the epithelial cells were arranged as crack structure with lymphocytes infiltrated sparsely. By immunohistochemical staining assay, the epithelial cells were positive for AE1/AE3 and CK19, and the lymphocytes were CD3 and CD20 positive. Based on the histological characters, a diagnosis of LFA was made, and the total follow-up period was determined to be forty six months. The final repeated CT scan revealed no recurring or residual lesions were detected during the post-surgical course.

**Virtual slide:**

The virtual slide(s) for this article can be found here: http://www.diagnosticpathology.diagnomx.eu/vs/1500429801911703

## Background

Lipofibroadenoma (LFA), a thymic tumor, was classified into “rare thymomas” in the WHO classification [1]. Until now, one and only case of LFA was reported in the English literature [2], in which a 62-year old man was suffered from dyspnea, dizziness and pure red cell aplasia. The tumor was found in the anterior mediastinum by Chest X-ray scan and diagnosed as LFA with thymoma type B1. Here we report another case of LFA, which is not accompanied with any type of thymoma. The clinical and pathological features are presented.

## Case presentation

### Clinical data

**Figure 1:**
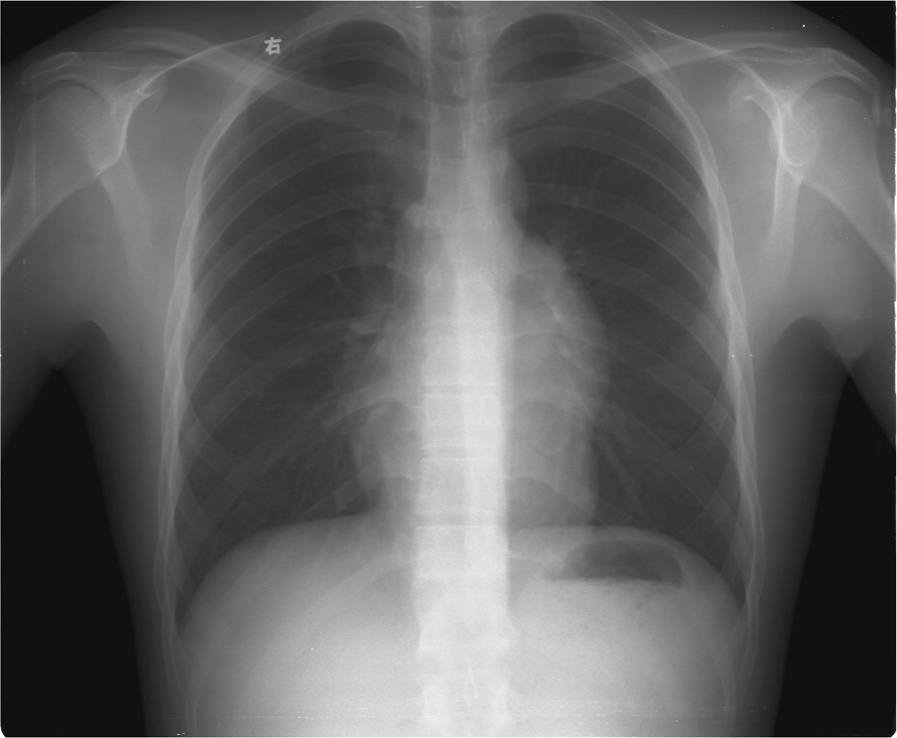
**Imaging.** Chest X-ray scan reveals a limited semicircle shadow located in the left heart edge.

The 21-year-old man was presented with a one-month history of mediastinal tumor, which was found during the regular medical examination in school, and he was then sent to our department of cardiothoracic surgery. Physical examination was negative for blepharoptosis, muscle weakness and palpable superficial lymph nodes, and the chest X-ray scan revealed a limited semicircle shadow located in the left heart edge (Figure 1). The blood counts assay, erythrocyte sedimentation rate, clinical blood biochemistry, blood urea nitrogen and serum creatinine, urine analysis, and endocrine profile were all within normal ranges.

### Macro pathology

**Figure 2:**
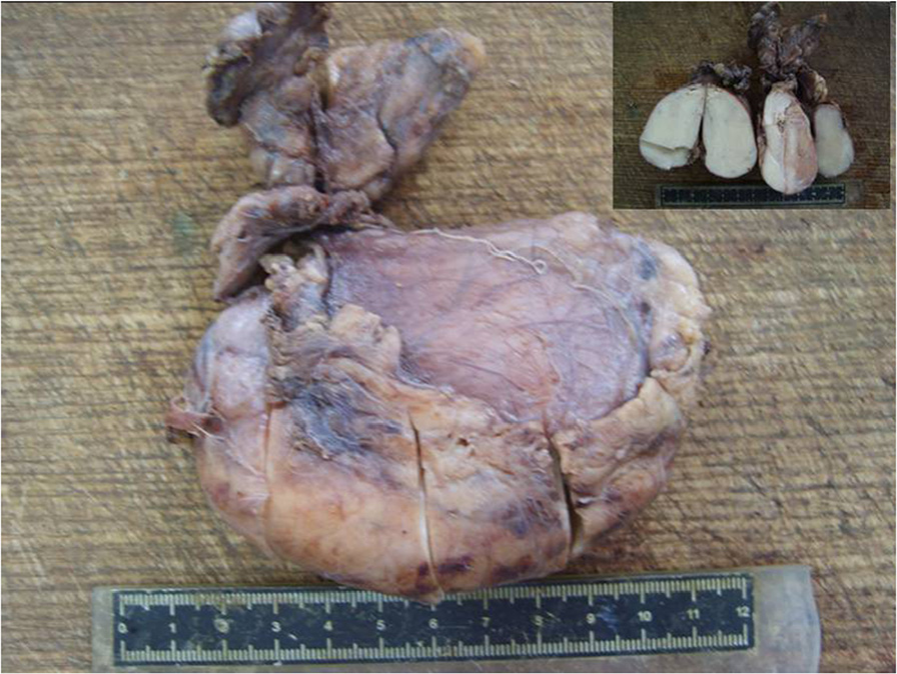
**Gross morphology of the tumor.** The tumor is oval with clear margin. Cut surface gives a solid appearance, grey in color and hard in quality (top right corner).

The surgery was performed under general anesthesia and supine position. An oval tumor in the anterior mediastinum, which was closely to the thymus, was observed during the procedure. The tumor and circumambient thymus were excised. In gross, the tumor was oval and with the volume of 10cm×6cm×4cm, in which the cut surface was a solid appearance, grey in color and hard in quality (Figure 2). The volume of remaining thymus was 6cm×4.5cm×2cm.

### Histology

**Figure 3:**
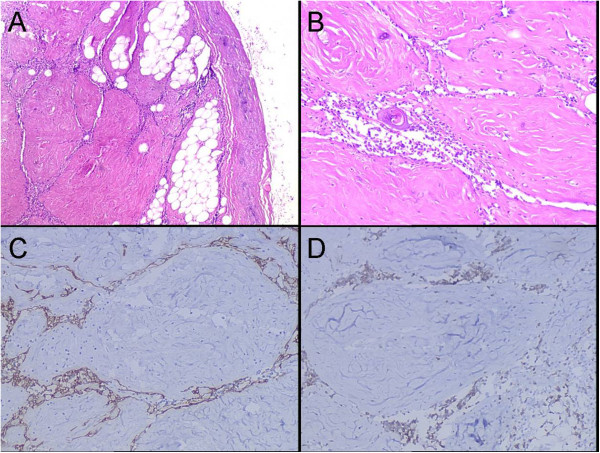
**Histological examination and immunohistochemical features of the tumor. (A)** The tumor shows irregularly connected strands forms a “Dog-like” structure in a dense fibrous tissue with scattered individual or groups of fat cells (H&E staining, ×40). **(B)** Sparse lymphocytes are infiltrated in the slender elongate epithelial strands and in some area, thymic corpuscle can be observed (H&E staining, ×100). **(C)** The epithelial strands of the tumor are positive for CK19 (Envision×100). **(D)** Lymphocytes are immunostained by CD3 (Envision×100).

Under the microscope, a clear boundary was shown between the tumor and the remaining thymus. The tumor was observed with irregularly connected figurate strands of thymic epithelial cells in a fibrous tissue, in which the fat cell was distributed singly or multifocally. The elongated epithelial were recorded to be formed various “animal-like” structures and sparse lymphocytes were infiltrated (Figure 3A). The epithelial cells were without obvious atypia and the mitosis was not observed. In some area, thymic corpuscle could be found (Figure 3B).

### Immunohistochemistry

Using immunohistochemical staining, the epithelial strands of the tumor were positive for AE1/AE3 and CK19 (Figure 3C), and the lymphocytes were immunostained with CD3 and CD20 (Figure 3D). The Ki67 labeling index was also calculated to be approximately less than 1%. Based on histological features and immunohistochemical characters, a diagnosis of LFA of the thymus was made, and the total follow-up period was determined to be forty-six months. The repeated X-ray scan revealed no recurring or residual lesion was found during the post-surgical course.

## Discussion

LFA is an unusual thymic tumor, and the clinical and pathological features are still unclear. Until now, only one case has been reported, and the clinical information is showed in table 1. The patient was a 62-year old man accompanied with pure red cell aplasia, and the clinical presentations were dyspnea and dizziness. Histologically, the tumor was accompanied with B1-type thymoma. The present case is a 21-year old man without any symptom. The pure red cell aplasia was not found and the tumor was not associated with any type of thymoma. The primary site of the tumors were all located in the anterior mediastinum, and both patients were received thymectomy and all alive with no evidence of disease after surgery.

**Table 1:** Previously reported cases of lipofibroadenoma of the thymus

**Series**	**Age (years)**	**Sex**	**Site primary**	**Clinical presentation**	**Accompanied with PRCA**	**Accompanied with other thymoma**	**Treatment**	**Size**	**Follow-up months/Status**
Kuo T et al. [2]	62	M	AM	Dyspnea and dizziness	Yes	Yes, Accompanied with B1-type thymoma	thymectomy	NA	80/ANED
Present case	21	M	AM	No symptom	NO	NO	thymectomy	10cm×6cm×4cm	46/ANED

Abbreviations: *NA* not available, *M* male, *AM* anterior mediastinum, *PRCA* pure red cell aplasia, *ANED* alive with no evidence of disease.

Pathology is still the gold standard in the diagnosis of LFA, in which the classic histological features were with thymic epithelial cells arranged as crack structure under the background of fibrous tissue. Lymphocytes are infiltrated in the crack and fat cells are distributed as individual or groups. In rare case, thymic corpuscle could be found. As far as the present case is concerned, the typical morphological characteristics are observed under the low power. Epithelial cells were positive to AE1/AE3, CK19 and the lymphocytes were immunostained with CD3 and CD20, which were used to make the diagnosis of LFA.

The differential diagnosis of LFA in histology primarily separated into thymolipoma and fibroadenoma, which intraductal type composed of interstitial and epithelial component. Epithelial cells were arranged as crack under the fibrous element, and the lack of fat cells and thymic component could be helpful in the distinguishing diagnosis. Thymolipoma was an unusual thymoma, which could lead to myasthenia gravis and autoimmune dysfunction [3]. Recent report suggested that thymolipoma origined from thymic true hyperplasia [4]. Under the microscope, epithelial and fibrous components can not be observed in the thymolipoma, which was the important point distinguishing from LFA. In addition, the biomarkers of CD57, c-Jun, p73, Casp9, and N-ras are also useful in the differential diagnosis [5,6].

The treatment for the patient we presented was thymectomy, and the necessary follow-up examination among forty-six months disclosed there was no palindromic lesion. RJ *et al.* had ever reported that COX-2 was expressed in all subtypes of thymomas and thymic carcinomas [7], which indicated COX-2 might be another potential novel target beside in thymic tumor therapeutic areas.

In general, LFA was a rare and benign thymic tumor, which might not be related to pure red cell aplasia, and the tumor accompanied with or without any type of thymoma. Histological features and immunohistochemical staining played an important role in diagnosis and differential diagnosis, in which thymolipoma and fibroadenoma were primary differential diagnosis. Thymectomy was regarded as the best treatment. However, much more cases are needed for further research.

## Consent

Written informed consent was obtained from the patient for publication of this case report and any accompanying images. A copy of the written consent is available for review by the Editor-in-Chief of this journal.

## Competing interests

The authors declare that they have no competing interests.

## Authors’ contributions

QG designed the study, performed the histological evaluation, and drafted the manuscript. QG and YG participated histological diagnosis and revising the manuscript. ZQ was involved in literature search and preparing the material. MJ and WX participated in providing the clinical information of this case. All authors read and approved the final manuscript.
